# LRMAHpan: a novel tool for multi-allelic HLA presentation prediction using Resnet-based and LSTM-based neural networks

**DOI:** 10.3389/fimmu.2024.1478201

**Published:** 2024-11-28

**Authors:** Xue Mi, Shaohao Li, Zheng Ye, Zhu Dai, Bo Ding, Bo Sun, Yang Shen, Zhongdang Xiao

**Affiliations:** ^1^ State Key Laboratory of Bioelectronics, School of Biological Science and Medical Engineering, Southeast University, Nanjing, China; ^2^ Department of Obstetrics and Gynecoloty, Zhongda Hospital, School of Medicine, Southeast University, Nanjing, China; ^3^ Jiangsu Sports Health Research Institute, Institute of Sports and Health, Nanjing, China

**Keywords:** biomedical engineering, neoantigen prediction, deep learning, multi allelic HLA, MHC, antigen processing

## Abstract

**Introduction:**

The identification of peptides eluted from HLA complexes by mass spectrometry (MS) can provide critical data for deep learning models of antigen presentation prediction and promote neoantigen vaccine design. A major challenge remains in determining which HLA allele eluted peptides correspond to.

**Methods:**

To address this, we present a tool for prediction of multiple allele (MA) presentation called LRMAHpan, which integrates LSTM network and ResNet_CA network for antigen processing and presentation prediction. We trained and tested the LRMAHpan BA (binding affinity) and the LRMAHpan AP (antigen processing) models using mass spectrometry data, subsequently combined them into the LRMAHpan PS (presentation score) model. Our approach is based on a novel pHLA encoding method that enables the integration of neoantigen prediction tasks into computer vision methods. This method aggregates MA data into a multichannel matrix and incorporates peptide sequences to efficiently capture binding signals.

**Results:**

LRMAHpan outperforms standard predictors such as NetMHCpan 4.1, MHCflurry 2.0, and TransPHLA in terms of positive predictive value (PPV) when applied to MA data. Additionally, it can accommodate peptides of variable lengths and predict HLA class I and II presentation. We also predicted neoantigens in a cohort of metastatic melanoma patients, identifying several shared neoantigens.

**Discussion:**

Our results demonstrate that LRMAHpan significantly improves the accuracy of antigen presentation predictions.

## Introduction

Peptide-HLA (pHLA) complexes consist of peptides that attach to human leukocyte antigens (HLA) and are presented to specialized immune cells, thereby initiating an immune response. HLA molecules are crucial for this process, as they present antigenic peptides on the cell surface for recognition by T cells (1, 2). This antigen presentation allows T cells to identify and attack infected or mutated cells. Infections can act as etiological factors in the development of various cancers. HLA molecules are integral to the anti-cancer immune response, playing key roles in the management of multiple cancer types, including lung, prostate, breast, and colon cancer (3–8).

HLA-I genes are highly polymorphic, with HLA heavy chains encoded by three genes: HLA-A, HLA-B, and HLA-C. All three genes are polymorphic, constituting the most distinctive feature of HLA molecules, which leads to variability in peptide presentation (typically 8-11 amino acids) among individuals (9, 10). Additionally, HLA-II molecules, located on human cells and consisting of three loci on chromosome 6 (DR, DQ and DP), are involved in the presentation of exogenous antigen (usually 13-25 amino acids) (11). The binding of peptides to HLA is the most critical and selective step in antigen presentation (12), making the identification of pHLA essential for developing effective immunotherapeutic cancer vaccines and studying infectious disease (13, 14). This highlights the need for in silico algorithms capable of accurately predicting pHLA molecules.

Several tools have been developed to address the challenges of neoantigen prediction, employing two main types of computational methods: single allele (SA) and multiple allele (MA) predictors. Both types typically consist of two predictive models: HLA-I binding affinity (BA) (15–18) and antigen processing (AP) (19–21) predictors. Recent advancements in mass spectrometry (MS) technology have facilitated the identification of peptides in high-throughput experiments, creating opportunities for developing neoantigen predictors. MHCflurry 2.0 (22) has integrated AP and BA predictors to significantly enhance prediction accuracy. Traditionally, published models segment MA mass spectrometry (MS) sequences into SA MS sequences for independent integration of pHLA into predictive models. Conversely, our approach directly integrates MA and peptides into the model as a cohesive entity, enhancing prediction accuracy through interactions between MA and peptides. Furthermore, combining peptide sequences with MA predictors (22, 23) offers greater intuitiveness and alignment with real human environments. However, studies utilizing multi-allelic (MA) data remain limited. Specifically, when considering the use of MA data as a whole input based on input patterns, the only available MA predictor is LRMAHpan.

ResNet (24) has been successfully applied in image recognition, yet the challenging of using ResNet for antigen presentation prediction has not been thoroughly explored. The shortcut connections of ResNet network significantly reduce the complexity of training deep neural networks (25, 26). The ResNet architecture consists of multiple similar residual blocks arranged in series. The Coordinate Attention (27) (CA) mechanism captures location and channel relationships, enabling the network to gather information from a larger area without significant resource consumption (28–30).

In this study, we address the limitations of preprocessing that arise from the one-to-one correspondence between peptide sequences and HLA types by utilizing ResNet_CA-based deep convolutional neural networks for the BA model and LSTM neural network for the AP model. LRMAHpan introduces a novel coding approach that utilizes 6-channel pHLA encoding as input data for residual networks, with each channel representing one of the six HLA types. LRMAHpan is the first ResNet_CA-based method for predicting antigen presentation, leveraging data from multiple allele (MA) mass spectrometry (MS) datasets to achieve accurate predictions. By incorporating a CA module, LRMAHpan effectively captures crucial binding signals directly from MA MS raw data, thus improving binding accuracy across various alleles and peptide sequences. The model can handle peptide sequences of variable lengths (8-11 amino acids), and we also trained and validated its performance in pHLA-II presentation by adjusting the number of channels and the length of peptide sequences (13-25 amino acids). Finally, we assembled different AP and BA predictors to forecast the potential of MA HLA in presenting sequences, resulting in the development of the presentation score (PS) (LRMAHpan PS). Our findings indicate that PS predictor outperforms both AP and BA models, demonstrating superior performance compared to commonly used tools such as NetMHCpan 4.1 (31), MHCflurry 2.0 and TransPHLA (32).

## Materials and methods

### Datasets

We used the multiple allele (MA) mass spectrometry (MS) datasets curated by EDGE (23), integrating them with an additional dataset derived from MHCflurry2.0 (22) to train the final version of our model. Negative samples were generated from peptides sourced from the reference proteome (SwissProt) that were not detected by mass spectrometry in the original samples. Specifically, we randomly sampled two segments from each negative peptide sequence, with the length of each segment reflecting the distribution of lengths in the positive dataset. From each sample, we randomly selected 1,800 data points, ensuring that no peptide sequences overlapped with those present in the positive dataset. Consequently, the final dataset maintained a 1:4 ratio of positive to negative samples, with a training set comprising 221,061 positive samples. HLA typing for the MA in the training set is detailed in **Supplementary Table S2**. Due to the frequent sharing of high-frequency alleles among patients, our analysis revealed a total of 118 unique HLA typing combinations, each associated with the presentation of more than 30 peptide sequences.

To mitigate variability associated with data preprocessing, we utilized existing post-processed training datasets to directly assess prediction systems (see **Supplementary Table S1**). The test dataset was obtained from MULTIALLELIC-RECENT benchmark dataset of MixMHCpred 2.0.2 (33), which includes mass spectrometry (MS) data from tumor samples of ten patients. The ratio of presenting peptides to non-presenting peptides in this dataset is 1:99. As predicted events (i.e., presenting peptides) are rare, achieving a high positive predictive value (PPV) becomes increasingly challenging, resulting in a more stringent evaluation of the model’s performance. To mitigate the impact of negative sample selection on the results, we also employed multi-allelic dataset provided by the IEDB database, which includes both presenting and non-presenting peptides, maintaining a 1:1 ratio for predictions (see **Supplementary Note S2**). Importantly, there is no overlap between the test and training datasets. In the training set, we excluded data from patients with incomplete HLA typing to enable the model to learn more accurate features of multi-allelic types. Consequently, the model prefers complete HLA data during predictions. If HLA typing information for a patient is incomplete at the time of prediction, our model can still process the data by supplementing it with the patient’s known typing information. Detailed usage instructions are available on GitHub and in **Supplementary Note S2**.

Data from cBioportal (34, 35) were retrieved from metastatic melanoma cohorts to predict neoantigens using LRMAHpan. The cohort was constructed by sequencing the whole exomes of 38 pairs of pre-treatment melanoma tumors and normal tissues. This data includes mutation maps in MAF format for 38 cases and RPKM expression data obtained from mRNA analysis for 27 cases. Additionally, the dataset includes results of HLA class I and class II typing.

### HLA representation

HLA typing was carried out using the OptiType 1.3.1 HLA analysis software packages. This tool was utilized to generate HLA types from matched normal DNA samples, allowing for accurate computational HLA typing. HLA class I alleles are represented by a “pseudo sequence” proposed by NetMHCpan (36). In our approach, we utilize the pseudo sequence generated by MHCflurry 2.0, which differs from the NetMHCpan pseudo sequence in that it has a length of 37. In addition to the 34 peptide contact positions contained in the NetMHCpan pseudo sequence, we incorporate three new positions (115, 126, and 23). These additional positions are selected to differentiate alleles that share the same NetMHCpan pseudo sequence. The pseudo-sequence of HLA class II is derived from the representation offered by NetMHCIIpan3.0 (37), which includes amino acid residues critical for peptide binding. It comprises 15 residues from the α chain and 19 residues from the β chain of HLA class II molecules, resulting in specific residues at defined positions. For the α chain, these positions are 9, 11, 22, 24, 31, 52, 53, 58, 59, 61, 65, 66, 68, 72, and 73. For the β chain, the positions are 9, 11, 13, 26, 28, 30, 47, 57, 67, 70, 71, 74, 77, 78, 81, 85, 86, 89, and 90. Consequently, the final length of the pseudo-sequence for HLA class II molecules totals 34 residues (15 from the α chain and 19 from the β chain).

### Peptide-HLA encoding

**Figure 1:**
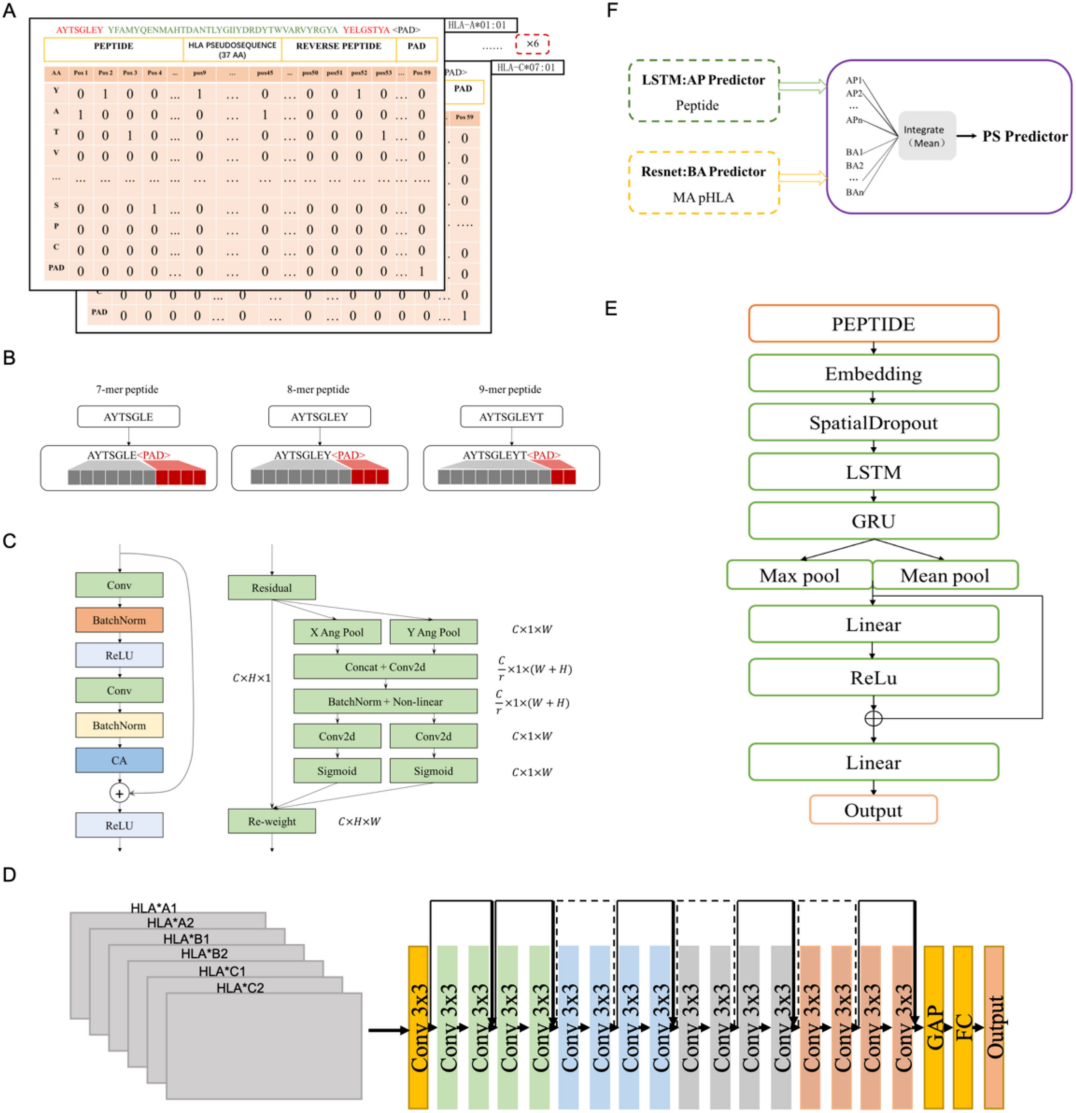
The structure of LRMAHpan PS predictor, LRMAHpan BA predictor and LRMAHpan AP predictor. **(A)** BA model input representations, for example, AYTSGLEY coding+ HLA pseudosequence coding+ YELGSTYA coding. Notably, each BA model input consists of six such data representations. **(B)** The input scheme accommodates peptides with variable lengths, capable of handling peptides of any length by selecting the maximum length, set here at 11. **(C)** The major sub-module (CA module) of BA predictor. **(D)** The LRMAHpan BA predictor adopts a Resnet structure. **(E)** The LRMAHpan AP predictor employs an LSTM structure. **(F)** The LRMAHpan PS predictor is proposed as a composite of two models **(D, E).** The LRMAHpan PS model is designed to predict neoantigens, combining multiple AP and BA models through the calculation of mean values.

The input to the LRMAHpan BA network was generated by scanning six HLA allele pseudosequences and peptide sequences (see **Figure 1A**). For each peptide sequence, a 3-dimensional feature matrix M (size 6×59×22) was constructed, comprising six channels of size 59×22, with each channel corresponding to one HLA allele type. This design aims to capture the signal from HLA peptide binding. Here, 59 represents the sum of the corresponding peptide length, pseudosequence length, and reverse peptide length, with all peptides padded to a maximum length of 59 using padding characters. Furthermore, 22 represents 20 common amino acids sequences, along with the padding marker <PAD>. Each amino acid in the peptide sequence was vectorized using a one-hot encoding scheme (20 common amino acids + <PAD>). Consequently, each allele peptide is represented by a two-dimensional vector of size (59, 22).

The peptide lengths range from 8 to 11 amino acids (AA), as this range encompasses about ninety-five percent of HLA class I presented peptides. The LSTM model employed the nn.Embedding module from PyTorch, which initializes embedding weights randomly. In the LRMAHpan AP mode, peptide sequences were vectorized using a parameterized embedding method, and peptides of multiple lengths (8-11 AA) were represented as vectors of fixed length by adding amino acid alphabets with padding characters and ensuring that all peptides were filled to a maximum length of 11 (see **Figure 1B**). For the HLA class II data, peptides with lengths ranging from 13 to 25 amino acids were included.

### The construction of neural networks

The LRMAHpan BA model incorporates a Coordinate Attention (CA) module into the ResNet residual block module (see **Figure 1C**). It accepts any intermediate feature tensor $X=\left[x_{1},x_{2},\ldots ,x_{C}\right]\in {\mathbb{R}}^{C\times H\times W}$ as input and outputs a transformed tensor with augmented representations $Y=\left[y_{1},y_{2},\ldots ,y_{C}\right]$ of the same size as X . To balance data volume and model size, we utilize a ResNet18 model comprising 17 convolutional layers and one fully connected layer, structured as follows (see **Figure 1D**). The input matrix is processed through the initial convolutional layer with a kernel size of 3×3, followed by four series of Residual Blocks, and then passed through AdaptiveAvgPool2d. The output of the final block is fed into a fully connected layer with an output size of 2, predicting whether the peptide can be presented by HLA.

The LRMAHpan BA model adds a CA module after the BatchNorm layer in the residual block module to enhance the feature representation. This CA approach addresses the challenge of location information loss from 2D global pooling by partitioning channel attentions into two parallel 1D signature encodings, effectively integrating spatial coordinate information into resultant attention maps. Notably, the CA technique features adaptability and a lightweight design, leveraging collected location data for precise region-of-interest capture and effectively capturing inter-channel relationships.

In channel attention mechanisms, global pooling is typically employed to comprehensively encode spatial information. However, this approach compresses global spatial data into a channel descriptor, which poses challenges in retaining positional information. To facilitate attention blocks in capturing distant spatial interactions with precise positional details, Coordinate Attention (CA) Blocks decompose global pooling into a pair of 1D feature encoding operations, as shown in Equation 1. Given input X, we utilize two spatial extents of pooling kernels (H, 1) or (1, W), to encode each channel along the horizontal and vertical coordinates, respectively. The squeeze step for the c-th channel can be expressed as follows:


(1) $$z_{c}=\frac{1}{H\times W}\sum_{i=1}^{H}\sum_{j=1}^{W}x_{c}\left(i,j\right).$$

Thus, the output of the c-th channel at height h can be formulated as:


(2) $$z_{c}^{h}\left(h\right)=\frac{1}{W}\sum_{0\leq i<W}x_{c}\left(h,i\right)$$

Similarly, the output of the c-th channel at width w can be expressed as:


(3) $$z_{c}^{w}\left(w\right)=\frac{1}{\text{H}}\sum_{0\leq j<H}x_{c}\left(j,w\right)$$

Here, $z_{c}$ denotes the output associated with the c-th channel. The input X is derived directly from a convolutional layer with a fixed kernel size, representing a set of local descriptors. The squeeze operation facilitates the aggregation of global information.

Upon obtaining the aggregated feature maps generated by Equations 2, 3, we concatenate them and pass them through a shared 1 × 1 convolutional transformation function F1, yielding:


(4) $$f=\delta \left(F_{1}\left(\left[z^{h},z^{w}\right]\right)\right).$$

where [·, ·] denotes the concatenation operation along the spatial dimension, δ is a non-linear activation function, and $f\in {\mathbb{R}}^{C/r\times \left(H+W\right)}$ is the intermediate feature map that encodes spatial information in both the horizontal and vertical directions. We then split f along the spatial dimension into two separate tensors $f^{h}\in {\mathbb{R}}^{C/r\times H}$ and $f^{w}\in {\mathbb{R}}^{C/r\times W}$ . Two additional 1 × 1 convolutional transformations, $F_{h}$ and $F_{w}$ , are utilized to separately transform $f^{h}$ and $f^{w}$ to tensors with the same channel number as the input X, yielding.


(5) $${ɡ}^{h}=\sigma \left(F_{h}\left(f^{h}\right)\right)$$

(6) $${ɡ}^{w}=\sigma \left(F_{w}\left(f^{w}\right)\right).$$

Here, σ is the sigmoid function, and the output of our coordinate attention block Y can be written as:


(7) $$y_{c}\left(i,j\right)=x_{c}\left(i,j\right)\times g_{c}^{h}\left(i\right)\times g_{c}^{w}\left(j\right).$$

To enhance the robustness and generalization capabilities of the BA model, our approach combines Sharpness-Aware Minimization (SAM) (38) and SGD (39) to achieve a balance between training duration and generalization capacity. Regardless of the gradient descent or optimization approach, the goal of training the model is to identify the parameters that minimize loss value. Notably, in contrast to other optimization methods, SAM achieves superior generalization by enhancing the training process through the simultaneous minimization of both loss value and loss sharpness. Furthermore, it explores parameters exclusively within neighborhoods exhibiting consistently low loss values, resulting in a flatter loss hyperplane compared to alternative optimization methods, thereby augmenting the model’s generalization capabilities. However, SAM requires double the training time due to computing the sharpness-aware gradient twice.

Based on the characteristics of the presented peptides, we propose a novel antigen peptide processing predictor based on the Bi-LSTM (40) framework, corresponding to LRMAHpan AP. The LRMAHpan AP predictor (see **Figure 1E**) comprises several key layers: embedding, spatial dropout, LSTM, GRU, Relu (41), maximum pooling, average pooling, and fully connected layers. SpatialDropout (42) randomly eliminates several feature dimensions. We utilize embedding-encoded peptide representations as the input to our model. Notably, the embedding dimension within the neural network is set at 100, while the hidden layers of both LSTM and GRU consist of 128 neurons each. Additionally, the largest pooling layer is connected to the average pooling layer to facilitate feature reuse, enhancing training efficiency and serving as input for subsequent layers.

This work introduces LRMAHpan PS as the ultimate presentation model, achieved by averaging the outcomes of LRMAHpan BA and LRMAHpan AP (see **Figure 1F**).

### Model training

For model training, we divided dataset (refer to the Dataets section) into multiple subsets: 95% for training and 5% for validation, utilizing different random seeds. A larger training dataset enables the model to learn more effectively and capture diverse binding patterns, which are crucial for its performance. The remaining 5% of the data is utilized as a validation set to evaluate the model’s performance and ensure its ability to generalize to unseen data. This approach aimed to identify the hyperparameters that minimize the loss value of the LRMAHpan BA model. We employed early stopping to monitor the performance metric, halting training when the performance on the validation set began to deteriorate. The neural network was trained using the SGD optimizer with a cross-entropy loss function. Training was conducted with a batch size of 128, an initial learning rate of 0.1 and a momentum value of 0.9. The learning rate was subsequently reduced to 0.02, 0.004, and 0.0008 at the 60th, 120th, and 180th iterations, respectively. The total training process encompassed 200 iterations. For optimizing the LRMAHpan AP model, we applied the same strategy. In this case, we divided the peptides into a training set (90%) and a validation set (10%), keeping all other training parameters consistent with those used for the LRMAHpan BA model.

### Model selection

The imbalance between positive and negative samples poses a significant challenge in tumor neoantigen prediction, potentially biasing model predictions towards the majority class. To address this issue, we employed an effective technique known as EasyEnsemble (43). This technique integrates undersampling and demonstrates strong performance in real-world scenarios. We set the ratio of positive to negative samples at 1:4, training the model with the sampled negative samples and all positive samples. The F1 score of the validation set was used as the performance metric for each model. Subsequently, we selected several top-performing models for ensemble averaging. The ensemble for LRMAHpan BA comprised nine models, while the ensemble for LRMAHpan AP included six models. During testing, the final prediction was generated by averaging the output probabilities from these selected models.

### Quantitative and statistical indicators

The model primarily employed PPV as the performance metrics, defined as PPV=NTP/(NTP+NFP), where NTP represents the number of true positives and NFP represents the number of false positives. The performance evaluation utilized Average Precision (AP) to assess the average precision and recall of a classification model at various thresholds. AP is particularly suitable for imbalanced datasets as it emphasizes the model’s ability to identify positive samples. For continuous PR curves, the formula for AP is given by:


(8) $$AP=\int_{0}^{1}PRdr$$

For discrete PR curves, the formula for AP is expressed as:


(9) $$\text{AP}=\sum_{k=1}^{n}P\left(k\right)\Delta R\left(k\right)$$

### Contrast with currently available tools

The purpose of this article is to evaluate LRMAHpan BA and LRMAHpan AP against the most advanced binding affinity predictor and presentation predictor (NetMHCPan 4.1, MHCflurry 2.0, TransPHLA). Our approach for assessing single allele (SA) predictors (NetMHCpan 4.1, MHCflurry 2.0, TransPHLA) using multiple allele (MA) data involves combining peptide sequences with each HLA typing separately, in accordance with the input characteristics of the predictors (see **Supplementary Note S1**). This method yields optimal results compared to our model (see **Supplementary Figure S3**).

The benchmark proposed by MHCflurry 2.0 was employed for performance comparison. To ensure a fair evaluation, the training dataset was omitted from the KESKIN MA dataset, as the MHCflurry 2.0 BA training process utilized a KESKIN SA cell line, which could provide an advantage in the MA dataset. Additionally, the exclusion of these datasets from the benchmark was motivated by the presence of the MULTIALLELIC-OLD data within the LRMAHpan training dataset. The final dataset used for comparison was a set of 10 datasets known as MULTIALLELIC_B, which contained a total of 18,472 presented peptides.

In the performance comparison, IC50 values were transformed into probability values ranging from 0 to 1. This facilitated comparisons between LRMAHpan Presentation Score (PS) and MHCflurry 2.0 PS, as well as between LRMAHpan BA and both MHCflurry 2.0 BA and NetMHCPan 4.1 BA. When comparing LRMAHpan AP with MHCflurry AP, it is important to highlight that MHCflurry 2.0 utilized a final training set comprising 493,473 MS data points and 219,596 affinity measurements, while LRMAHpan relied on only 221,061 presented MS data points. This indicates that our model can extract accurate features and make precise predictions using limited data.

## Results

### Prediction performance of LRMAHpan BA

**Figure 2:**
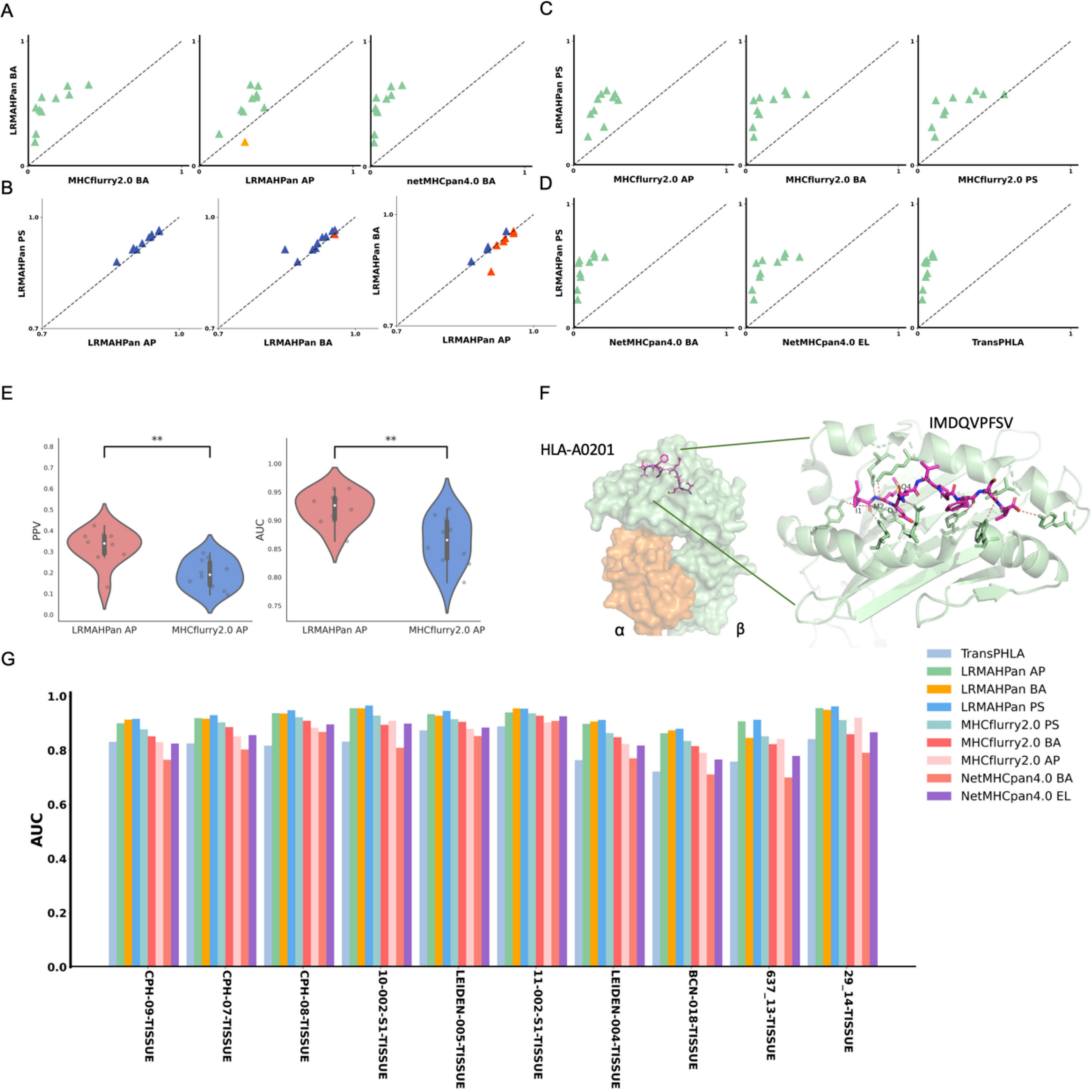
Benchmarking the performance of the models. **(A)** Performance comparison of PPV of the BA models against other predictors, with each point representing a single experiment. **(B)** AUC values of LRMAHpan. **(C, D)** PPV of LRMAHpan PS is contrasted with other predictors. **(E)** Violin plot display PPV and AUC values of LRMAHpan AP alongside MHCflurry2.0 AP across ten independent test sets. **(F)** The structural depiction of the experimental complex involving the epitope IMDQVPFSV presented by HLA-A:02*01, showcasing detailed residue interactions. The α and β chains within the HLA-A:02*01 structure are highlighted in green and orange, respectively, while non-covalent interactions between HLA and peptide residues are illustrated by dashed lines using PyMOL. **(G)** AUC values of 9 models on 10 independent test sets. The symbol ** indicates that the p-value is less than 0.01.

To evaluate the performance of the LRMAHpan BA predictor based on the ResNet_CA network, we screened ten samples from MULTIALLELIC benchmark (see Methods for more details), ensuring the inclusion of six HLA alleles as an independent test dataset (**Table 1**). Each peptide sequence was combined with an HLA pseudosequence and a reverse peptide sequence, then encoded into a vector (**Figure 1A**). Additionally, each peptide sequence could be separately combined with six HLA typings to form a six-channel data input for training and testing. A benchmark was established using public datasets of HLA ligands identified by mass spectrometry (MS) (**Supplementary Table S1**). We compared the performance of our model to that of the current state-of-the-art methods, MHCflurry2.0 BA and NetMHCpan4.1 BA (**Figure 2A**), which are widely used for predicting HLA ligands. LRMAHpan BA demonstrated superior performance compared to both MHCflurry2.0 BA and NetMHCpan4.1 BA when applied to test data. The positive predictive value (PPV) was calculated at the recall rate was 50% on a test set composed of ten subsets with a 1:99 ratio of positive to negative samples. For instance, the PPV of LRMAHpan BA, MHCflurry2.0 BA, and Netmhcpan4.1 BA were 0.477, 0.151 and 0.080, respectively (see **Supplementary Figure S1A**). In the dataset 29_14-TISSUE, which contained the largest number of positive samples, the PPV of LRMAHpan BA was 8.9 times higher than that of MHCflurry2.0 BA and 13.9 times higher than that of NetMHCpan4.1 BA (see **Supplementary Figures S1B, G**). To assess whether this advantage was consistent across different datasets, we tested data with positive to negative ratios of 1:1 and 1:9. The results indicated that regardless of the ratio, our PPV values were superior to those of existing tools (see **Supplementary Figure S1F**). Similarly, in dataset 637-13-TISSUE, the PPV of LRMAHpan BA was 4.1 times higher than that of MHCflurry2.0 BA and 7.9 times higher than that of NetMHCpan4.1 BA (see **Supplementary Figures S1C, G**). The excellent performance of the BA model in terms of PPV may be attributed to the multi-allelic model’s ability to recalled fewer false positive prediction predictions under the same datasets. Despite undergoing identical validation procedures, our model is unique in simultaneously considering six alleles, whereas the SA predictor necessitates multiple iterations involving peptide sequences and HLA typing six times. This distinction may result in higher recall rates for SA predictors, along with an increased likelihood of false positives.

**Table 1:** Independent test sets.

Sample id	#Pos	#Neg	HLA
11-002-S1-TISSUE	946	93654	A0301 A2402 B3503 B4402 C1203 C1203
10-002-S1-TISSUE	431	42669	A0201 A3101 B1302 B5801 C0602 C0701
BCN-018-TISSUE	935	92565	A0201 A2901 B0702 B2705 C0102 C1505
CPH-09-TISSUE	1527	151173	A0201 A3201 B2705 B4402 C0501 C0202
CPH-07-TISSUE	1816	179784	A0201 A0201 B3501 B2705 C0202 C0401
29-14-TISSUE	4049	400851	A0201 A3201 B4001 B1302 C0304 C0602
637-13-TISSUE	2386	236214	A0101 A2402 B5101 B0801 C0701 C0102
LEIDEN-005-TISSUE	2066	204534	A0201 A2501 B3501 B1801 C1203 C0401
CPH-08-TISSUE	3008	297792	A3201 A2601 B3801 B4002 C0202 C1203
LEIDEN-004-TISSUE	1308	129492	A0301 A0201 B0702 B0702 C1203 C0702

In addition, we trained a series of models and found that using ResNet with CA resulted in slightly higher performance compared to models without CA (**Table 2**), demonstrating an average improvement of 0.02 in the area under the curve (AUC). The PPV and AUC values of LRMAHpan BA across the ten test sets were consistently higher than those of MHCflurry2.0 BA and NetMHCPan4.1 BA (see **Figures 2A, G**; **Supplementary Figures S5A, B**). These observations illustrate that LRMAHpan BA possesses powerful feature extraction capabilities, generalizability and advantages in large datasets. Overall, LRMAHpan BA significantly improved predictive performance for MA presentation.

**Table 2:** Compare the AUC with and without CA module in the test sets of the BA predictor.

Sample id	ResNet18	ResNet18_CA
CPH-09-TISSUE	0.90	0.92 (+0.02)
CPH-07-TISSUE	0.91	0.93 (+0.02)
CPH-08-TISSUE	0.93	0.95 (+0.02)
10-002-S1-TISSUE	0.95	0.97 (+0.02)
LEIDEN-005-TISSUE	0.92	0.95 (+0.03)
11-002-S1-TISSUE	0.93	0.95 (+0.02)
LEIDEN-004-TISSUE	0.89	0.91 (+0.02)
BCN-018-TISSUE	0.86	0.88 (+0.02)
637_13-TISSUE	0.90	0.91 (+0.01)
29_14-TISSUE	0.95	0.96 (+0.01)

### Prediction performance of LRMAHpan AP

Comparing the predictive capabilities of LRMAHpan AP and LRMAHpan BA reveals some interesting insights. The LRMAHpan AP predictor outperforms MHCflurry 2.0 AP predictor in terms of PPV and AUC indicators (see **Figure 2E**; **Supplementary Figures S5C**). To investigate whether the AP predictor differs from the BA predictor in feature extraction, we evaluated LRMAHpan AP and LRMAHpan BA models using ten test sets from the MLTIALLELIC_B dataset (see **Figures 2A, B**). LRMAHpan AP model solely utilizes mass spectrometry-derived peptide sequences as input (see **Supplementary Figure S3**), while LRMAHpan BA model integrates data from six HLA types along with peptide sequences (see **Figures 1A, D**).

Interestingly, nine out of ten LRMAHpan BA samples exhibited higher PPV compared to LRMAHpan AP (see **Figure 2A**). Regarding AUC values, the LRMAHpan AP outperformed the LRMAHpan BA in six out of ten samples (see **Figures 2B**; **Supplementary Figures S5D**). The mean AUC of LRMAHpan AP predictor reached 0.92 (0.86-0.96), suggesting that the AP model effectively captures meaningful signals. Further comparisons of Recall, Accuracy, and F1 value on independent test sets highlight performance differences between the LRMAHpan AP and the LRMAHpan BA models (see **Supplementary Figure S2**). The LRMAHpan BA demonstrates higher accuracy and F1 score, while the LRMAHpan AP shows a higher recall rate.

Overall, LRMAHpan BA outperforms LRMAHpan AP in terms of predictive performance, partially attributed to the use of MA data and an improved approach for encoding peptide sequences. This enhanced performance can be attributed not only to the features of the training dataset (HLA-presented peptides) but also to the overall model design. The new model framework allows for learning connections between multiple alleles, rather than being limited to a single allele. In contrast, our AP model, which combines LSTM and GRU layers, exhibits a slight improvement in performance (see **Supplementary Figure S7**).

### Prediction performance of LRMAHpan PS

Furthermore, we explored whether the combination of the LRMAHpan AP and the LRMAHpan BA predictors could achieve superior prediction results. We subsequently compared the LRMAHpan PS model with several others, including MHCflurry 2.0 AP, MHCflurry 2.0 BA, MHCflurry 2.0 PS, NetMHCpan 4.1 EL, NetMHCpan 4.1 BA, and TransPHLA (see **Figures 2C, D**; **Supplementary Figures S5E, F**). The LRMAHpan PS exhibits an average PPV higher than those of MHCflurry 2.0 PS, NetMHCpan 4.1 EL, and TransPHLA, with values of 0.4747, 0.2615, 0.1534, and 0.0642, respectively (see **Supplementary Figure S1D**). Across all samples, LRMAHpan PS shows higher AUC values compared to MHCflurry 2.0 PS, NetMHCpan 4.1 EL, and TransPHLA, achieving values of 0.9329, 0.8947, 0.8518, and 0.8157, respectively (see **Supplementary Figure S1E**).

In comparison with MHCnuggets (44) and MixMHCPred, LRMAHpan exhibited superior performance across both AUC and AP metrics, as illustrated in **Supplementary Figure S6**. We tested our model on well-studied HLA-peptide samples, such as the IMDQVPFSV epitope presented by HLA-A*02:01, demonstrating that LRMAHpan accurately predicts the potential presentation of this peptide by the patient’s HLA (see **Supplementary Figure S4A**). Correlation analysis with HLA typing (see **Supplementary Figure S4B**) using the PSSM matrix (see **Supplementary Figure S4C**) indicates that IMDQVPFSV can be presented by either HLA-A*02:01 or HLA-C*0501. Experimental data further confirm the binding of IMDQVPFSV and HLA-A*02:01, providing additional evidence for the reliability of our model (see **Figure 2F**).

**Figure 3:**
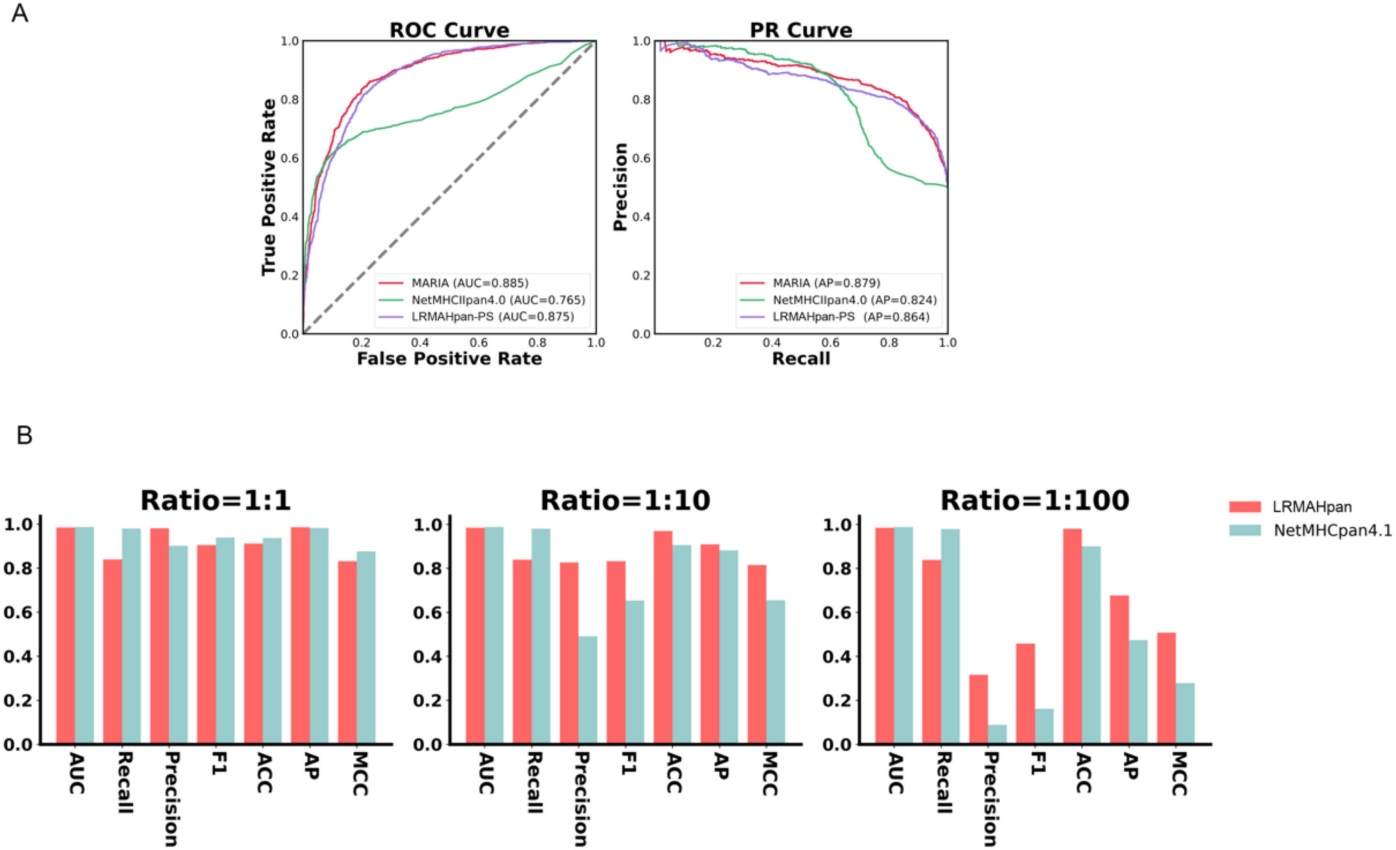
Generalization and robustness validation results. **(A)** Performance of PS model in predicting pHLA-II. **(B)** In K562 cell lines, Comparison of AUC, Recall, Precision, F1, ACC, AP and MCC values of LRMAHpan PS and NetMHCpan4.1 with positive and negative sample ratios of 1:1, 1:10 and 1:100, respectively.

To assess the robustness of our model, we obtained mass spectrometry data for an ovarian cancer patient from Dao (45), encompassing a total of 1,874 presentation instances. The HLA typing included HLA-A*02:01/A*01:01, HLA-B*57:01/B*07:05, and HLA-C*06:02/C*15:05. We evaluated LRMAHpan PS and NetMHCpan 4.1 using various performance metrics—AUC, Recall, Precision, F1, ACC, AP, and Matthews correlation coefficient (MCC) —across different positive-to-negative sample ratios (1:1, 1:10, and 1:100), as illustrated in **Figure 3B**. These metrics serve distinct purposes, with AUC and AP values providing threshold-independent evaluations. NetMHCpan 4.1 performs admirably at a positive-to-negative sample ratio of 1:1 but exhibits a significant drop in accuracy as the proportion of negative samples increases. In contrast, LRMAHpan PS demonstrates commendable performance in terms of precision, F1, ACC, AP, and MCC. Notably, at a positive-to-negative sample ratio of 1:100, LRMAHpan PS is poised to predict more authentic neoantigens due to its higher precision and AP values. Given the inherent imbalance between presented and non-presented antigens in real human settings, with non-presented antigens typically outnumbering presented ones, the performance metrics at a ratio of 1:100 are more reflective of real-world scenarios. This underscores the robustness and fidelity of our model predictions.

### Class II model proof of concept

We evaluated whether the prediction model we proposed can also be applied to class II HLA peptide presentation. We utilized class II mass spectrometry data from the MARIA (46) dataset, where each peptide corresponds to two HLA class II alleles, both expressing HLA-DRB1. The preprocessing steps included data deduplication, after which the dataset was divided into training and validation subsets. The AUC and AP values of the validation set were used as evaluation criteria. The model architecture and training methodology were consistent with those employed for predicting HLA-I peptide presentation, with the notable exception of incorporating two channels. Next, we evaluated the performance of LRMAHpan PS against the K562 DRB1*01:01 benchmark dataset from MARIA. which comprised 1,361 positive and 1,361 negative samples. We plotted the ROC and PR curves for the MARIA, NetMHCIIpan 4.0, and LRMAHpan PS, calculating their respective AUC and AP values. The results were 0.885 and 0.879 for MARIA, 0.765 and 0.824 for NetMHCIIpan 4.0, and 0.875 and 0.864 for LRMAHpan PS, respectively (see **Figure 3A**). Comparative analysis reveals that the AUC and AP values of MARIA and LRMAHpan PS exceed those of NetMHCIIpan 4.0, with LRMAHpan PS demonstrating comparable efficacy to MARIA. These findings underscore the robust generalization and migratory capabilities of our model framework.

### Examples of neoantigen prediction in metastatic melanoma cohorts

**Figure 4:**
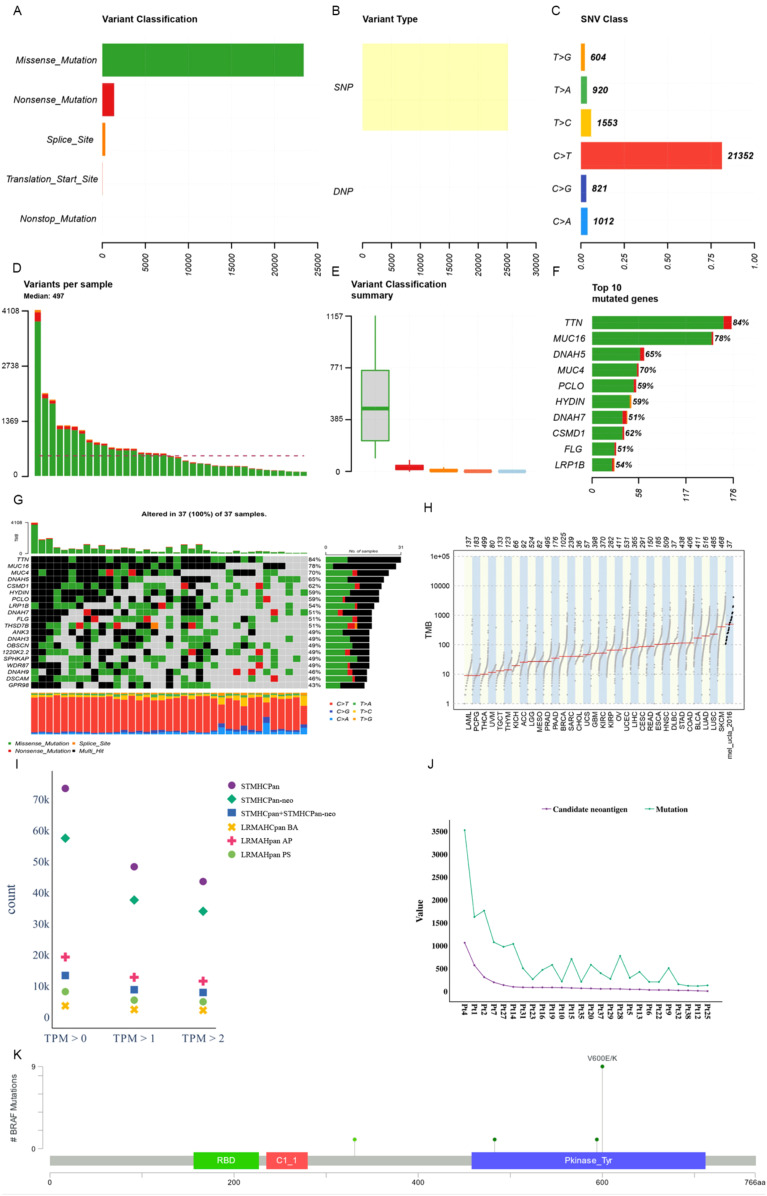
Mutational landscape of a metastatic melanoma cohort. **(A)** Overall variant classification by cohort. **(B)** Overall variant type by cohort. **(C)** Type of single nucleotide variation. **(D)** Number of variants per sample. **(E)** Cohort variant classification profile. **(F)** Top ten genes with the largest number of mutations. **(G)** Mutant landscape waterfall plot where multi_Hit indicates genes mutated more than once in the same sample. **(H)** Comparison to mutational load in a cohort of 33 cancer species already available in TCGA. **(I)** Distribution of antigen presentation quantities predicted by STMHCPan, STMHCPan-neo, STMHCPan+STMHCPan-neo, LRMAHPan BA, LRMAHPan AP, and LRMAHPan PS under TPM>0, TPM>1, and TPM>2. **(J)** The number of Candidate neoantigen predicted by the model compared to the number of SNV mutations per sample. **(K)** BRAF mutation distribution and protein domain in metastatic melanoma cohort.

We utilized Maftools (47) to visualize the cohort and assess the mutation status of all metastatic melanoma samples. The primary categorization of variations included missense mutation, with single nucleotide polymorphisms (SNPs) being the predominant variation type, characterized notably by the frequent occurrence of C > T transitions. Each sample exhibited significant variability in mutation burden, with a median of 497 mutations. TTN (84%) and MUC16 (78%) emerged as genes with substantial mutational frequencies (see **Figures 4A–F**). The waterfall plot demonstrates that some genes were altered multiple times across different samples (see **Figure 4G**). Comparing the mutation burden of metastatic melanoma to 33 other cancers in the TCGA revealed a notably high mutation load in melanoma (see **Figure 4H**).

Antigen presentation prediction was performed using 26 samples with available RNA expression levels employing LRMAHpan. Within the metastatic melanoma cohort, 14,462 single nucleotide variants (SNVs) were identified. Following segmentation around the mutation sites into 8-11mers, a total of 541,783 peptides were generated. The distribution of predicted peptides using STMHCPan (48), STMHCPan-neo, STMHCPan + STMHCPan-neo, LRMAHpan BA, LRMAHpan AP, and LRMAHpan PS was assessed under the conditions of TPM > 0, TPM > 1 and TPM > 2 (see **Figure 4I**). As TPM thresholds increased, the number of predicted peptides decreased. Specifically, under TPM>0, LRMAHpan PS projected 8,155 presented peptides; under TPM>1, 5,432 peptides were predicted; and under TPM>2, 4,905 peptides were anticipated. In the prediction of presented peptides in tumor patients, combining TPM with LRMAHpan significantly reduced the false positive rate. The number of predicted novel antigens for each sample correlated with the respective SNV mutation burden (see **Figure 4J**), suggesting that patients with a higher mutation load may derive greater benefit from immunotherapy targeting neoantigens.

We observed that most of the mutant peptides are unique, which may be related to the genetic diversity within the tumor and the high mutation load of melanoma. However, some shared neoantigens were detected, indicating peptide presentation across multiple samples. Specifically, LRMAHpan PS predicted 10 peptides to be presented in more than two samples (see **Table 3**), and 116 peptides were predicted to be presented by LRMAHpan PS in more than one sample.

**Table 3:** Shared neoantigen peptides with more than 2 samples.

Peptide	Sample Id	HGVSp_Short	Hugo_Symbol
AVYPRAGRK	Pt1/Pt16/Pt29	p.S381R	OAS3
SESTQENNQGY	Pt1/Pt4/Pt27	p.G444E	EBF2
AQVGVATY	Pt4/Pt6/Pt13	p.R381Q	VWA2
RAQVGVATY	Pt4/Pt6/Pt13	p.R381Q	VWA2
SRAQVGVATY	Pt4/Pt6/Pt13	p.R381Q	VWA2
KIGDFGLATEK	Pt6/Pt13/Pt14/Pt22/Pt32/Pt38	p.V600E	BRAF
VQDHGQPSL	Pt4/Pt16/Pt19	p.P684S/p.P653S	PCDHGA4/PCDHGA12
FLDPADIAA	Pt20/Pt28/Pt35	p.T315A	FAM160B2
FLDPADIAAL	Pt20/Pt28/Pt35	p.T315A	FAM160B2
ATDGGGLSEK	Pt5/Pt27/Pt35	p.E137K/p.G328E	PCDHB5/PCDHB6

Notably, LRMAHpan PS identified the peptide KIGDFGLATEK, derived from the BRAF V600E mutation, in six samples. The oncogenic BRAF mutation, found in approximately 40% of melanomas, leads to sustained activation of the MAPK signaling pathway, influencing tumor cell differentiation, proliferation, and metabolism (49). The BRAF V600E mutation, situated within the protein tyrosine kinase domain, was detected in 7 out of 26 samples within the metastatic melanoma cohort (see **Figure 4K**).

## Discussion

The prediction of antigen presentation is a pivotal aspect of anticipation tumor neoantigens. While many models for antigen presentation prediction predominantly concentrate on a single allele, the clinical dataset primarily consists of multi-allelic (MA) peptide sequences. As MA data continues to accumulate, direct MA antigen presentation prediction becomes feasible.

This study outlines strategies for applying ResNet in bioinformatics, specifically for predicting HLA class I peptide binding and presentation. By leveraging existing MA MS sequence encoding, we devised a representation conducive to integrating bioinformatics tasks with computer vision techniques. Utilizing this coding representation, we developed a ResNet-based architecture for HLA class I peptide binding prediction, which also yielded commendable results in predicting HLA class II binding. Notably, our framework enables the accurate prediction of any MA subtype. Despite being constructed with minimal data, our experimental findings on benchmark datasets demonstrate that our approach achieves state-of-the-art prediction performance across the majority of test sets compared to current models, particularly excelling on large datasets.

Initially, we explored data augmentation techniques to enhance the generalization ability of model. This approach was intended to increase the variability of the training data and improve performance on unseen samples. However, extensive validation revealed that the presence or absence of the data augmentation module had minimal impact on the overall performance of our predictive model. Consequently, we decided to remove the data augmentation step to streamline the computational process without sacrificing predictive accuracy.

Nevertheless, our model has limitations. In cases where HLA of patient type is incomplete, it requires supplementation based on known HLA typing of the patient, which may lead to some loss of accuracy. Additionally, our model’s capacity to predict neoantigens is restricted, as our work primarily focuses on HLA class I ligand presentation without verifying ligand binding to T-cell receptors (TCR). Future research will explore the potential integration of these predictors with TCR assessments.

## Data Availability

The datasets presented in this study can be found in onlinerepositories. The names of the repository/repositories and accession number(s) can be found in the article/**Supplementary Material**.
