# Supplementary material for: LRMAHpan: a novel tool for multi-allelic HLA presentation prediction using Resnet-based and LSTM-based neural networks
Source: Front Immunol. 2024 Nov 28;15:1478201. doi: 10.3389/fimmu.2024.1478201 (PMC11634944; doi:10.3389/fimmu.2024.1478201)
Supplement: Supplementary file 1 [file DataSheet1.pdf]

Supplemental Information

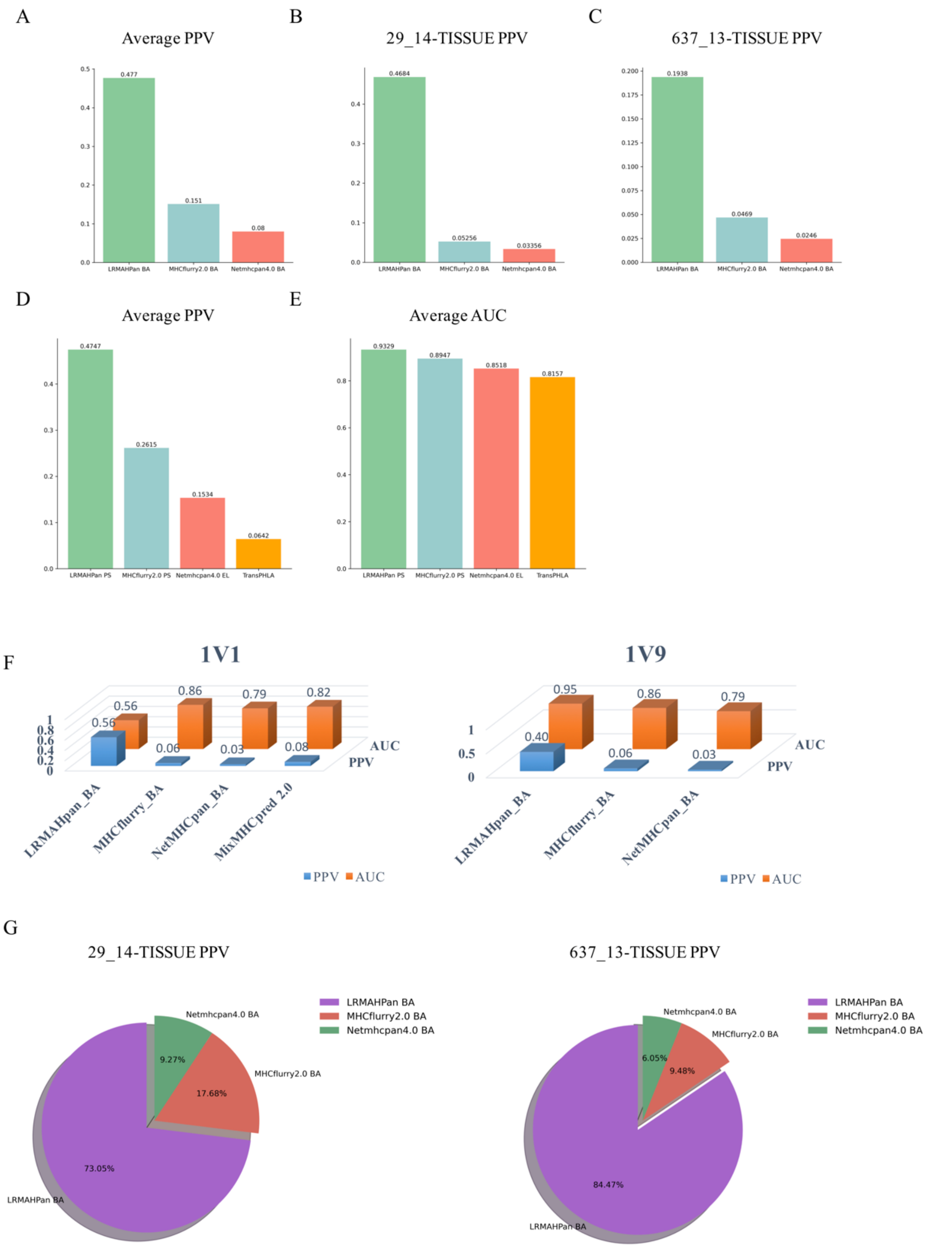

Figure S1 **AUC and PPV(recall=0.5) of LRMAHpan, MHCflurry2.0, Netmhcpan4.1 and TransPHLA on independent test sets.** (A) The average PPV of three BA models in all 10 test sets. (B) The PPV of three BA models in the dataset 29_14-TISSUE. (C) The PPV of three BA models in the dataset 637-13-TISSUE. (D) The average PPV of LRMAHpan PS, MHCflurry2.0 PS, NetMHCpan4.1 EL and TransPHLA in all 10 test sets. (E) The average AUC values of LRMAHpan PS,

MHCflurry2.0 PS, NetMHCpan4.1 EL and TransPHLA in all 10 test sets. (F) Indicators for evaluating positive-negative ratios of 1:1 and 1:9 for each model in the dataset 29_14-TISSUE. (G) Pie charts represent the proportions of the predicted performance of the three BA models in the dataset 29_14-TISSUE and 637-13-TISSUE.

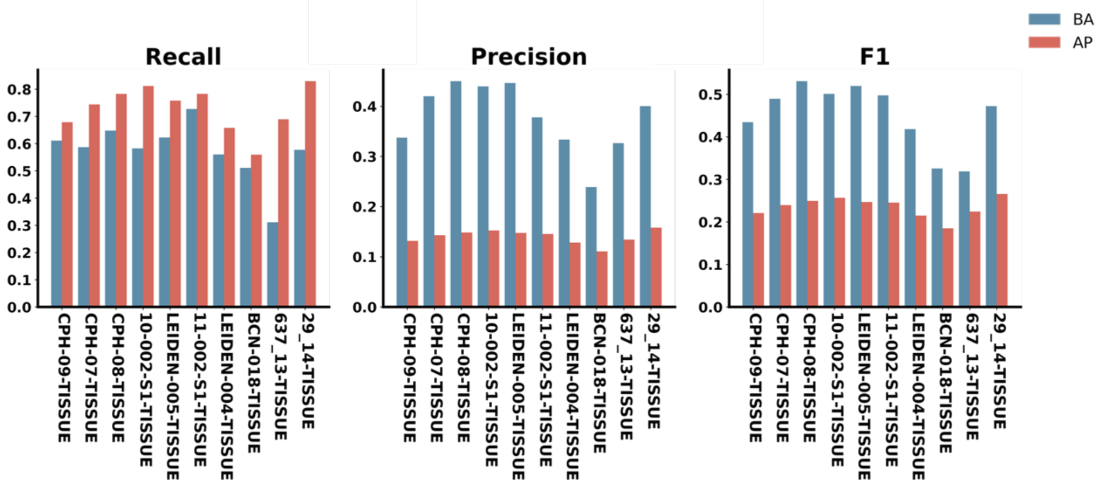

Figure S2 Recall, Precision and F1 scores of LRMAHpan AP and LRMAHpan BA model in all 10 test sets.

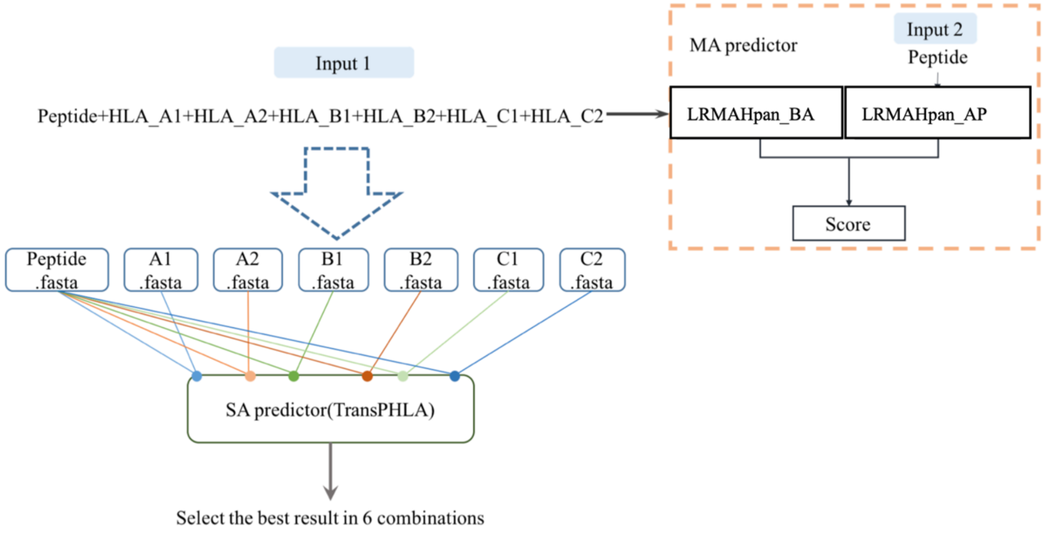

Figure S3 Input strategy for SA predictors and MA predictors.

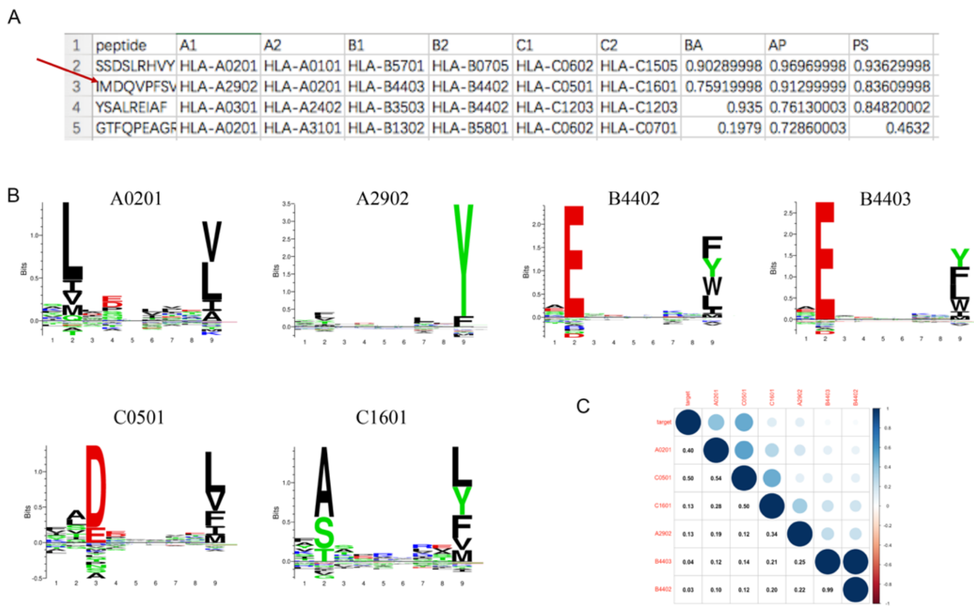

1	peptide	A1	A2	B1	B2	C1	C2	BA	AP	PS
2	SSDSLRHVY	HLA-A0201	HLA-A0101	HLA-B5701	HLA-B0705	HLA-C0602	HLA-C1505	0.90289998	0.96969998	0.93629998
3	IMDQVPFSV	HLA-A2902	HLA-A0201	HLA-B4403	HLA-B4402	HLA-C0501	HLA-C1601	0.75919998	0.91299999	0.83609998
4	YSALREIAF	HLA-A0301	HLA-A2402	HLA-B3503	HLA-B4402	HLA-C1203	HLA-C1203	0.935	0.76130003	0.84820002
5	GTFQPEAGF	HLA-A0201	HLA-A3101	HLA-B1302	HLA-B5801	HLA-C0602	HLA-C0701	0.1979	0.72860003	0.4632

Figure S4 **Example of using LRMAHpan prediction.** (A) The result shows that IMDQVPFSV can be presented by HLA-A:02*01. (B) The motif of HLA-A0201, HLA-A2902, HLA-A4402, HLA-A4403, HLA-C0501, HLA-C1601. These six subtypes originate from the same patient. (C) We conducted correlation analysis with HLA typing in (B) using the PSSM matrix.

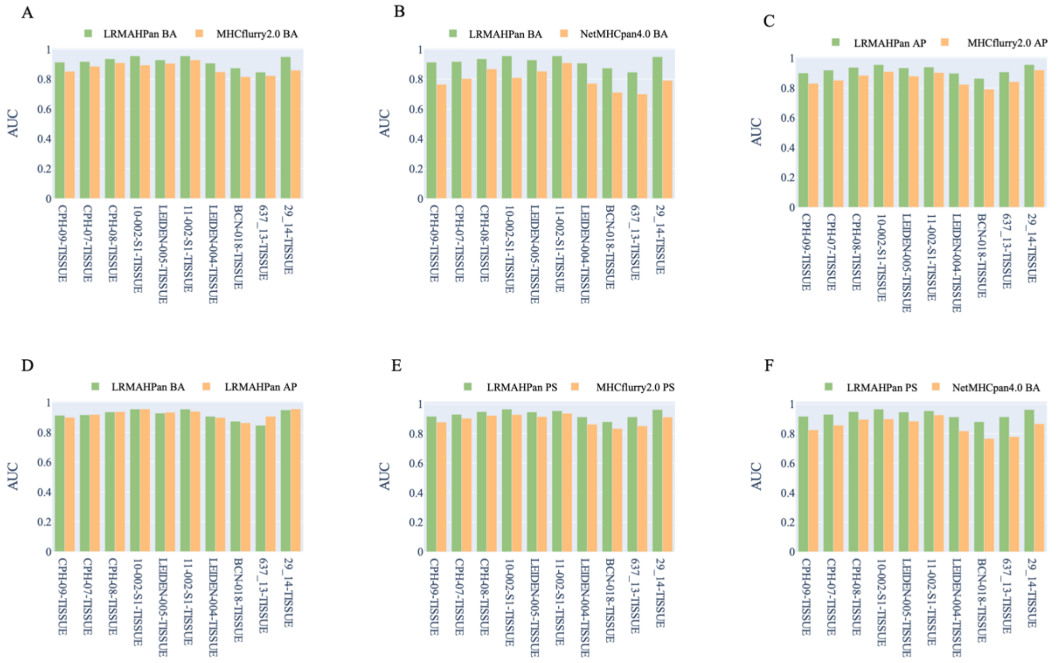

Figure S5 **AUC of LRMAHpan, MHCflurry2.0, NetMHCpan4.0 on independent test sets.** (A) AUC values of LRMAHpan BA and MHCflurry2.0 BA. (B) AUC values of LRMAHpan BA and NetMHCpan4.0 BA. (C) AUC values of LRMAHpan AP and MHCflurry2.0 AP. (D) AUC values of LRMAHpan BA and LRMAHpan AP. (E) AUC values of LRMAHpan PS and MHCflurry2.0 PS. (F) AUC values of LRMAHpan PS

and NetMHCpan4.0 BA.

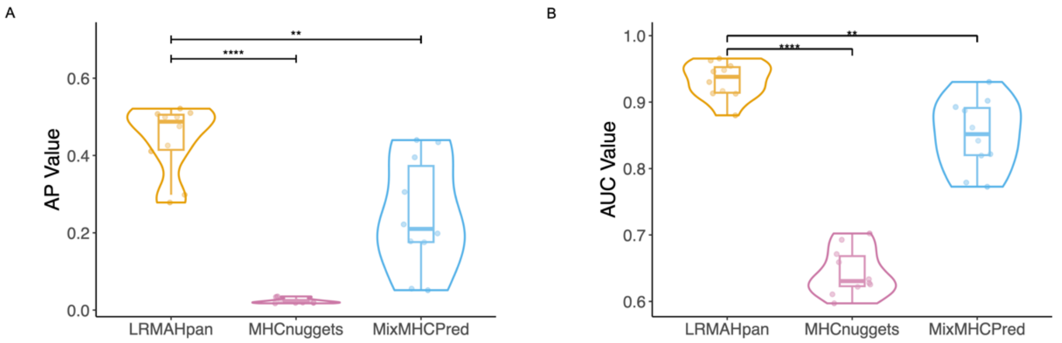

Figure S6 **AUC and AP values of LRMAHpan, MHCnuggets and MixMHCPred on ten independent test sets.** (A) Depicts a violin plot showcasing the distribution of AP values. (B) Displays a violin plot illustrating the distribution of AUC values.

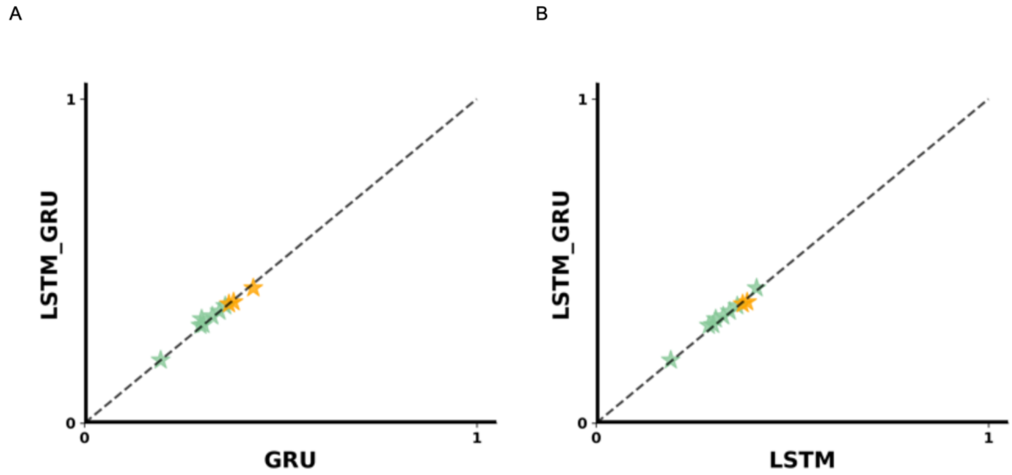

Figure S7: Performance comparison of PPV of LSTM_GRU against GRU and LSTM, with each point representing a single experiment. The green dots represent superior results for LSTM_GRU.

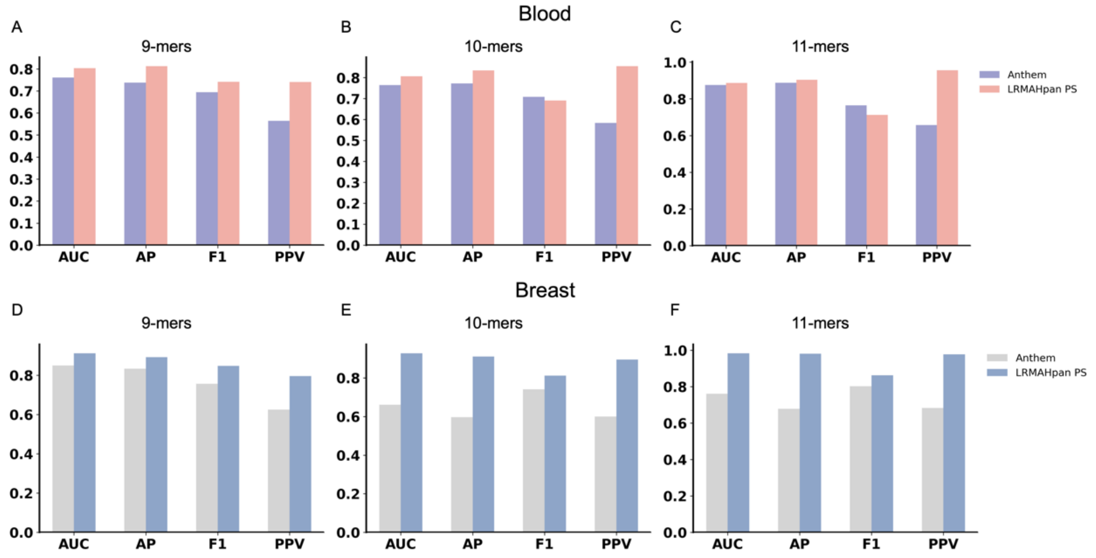

Figure S8: AUC, AP, F1, and PPV values of LRMAHpan PS and Anthem were evaluated on Blood and Breast test sets from IEDB for peptide lengths of 9-11.

Supplementary Note 1: Steps for Comparing Model Benchmarks

Step 1: Data Preprocessing

Prepare the data to meet the input requirements of each tool (NetMHCpan 4.1, MHCflurry 2.0, MHCnuggets, MixMHCPred, and Anthem). This involves transforming the test dataset into a compatible format for each model.

Step 2: Tool Prediction

Set up the environment according to the guidelines for each tool, using the preprocessed data from Step 1 to generate predictions.

Step 3: Result Processing

As each model predicts HLA types separately using peptide sequences, we will retain the optimal value from the six allele prediction results for comparison with LRMAHpan.

Supplementary Note 2: Comparison the computational footprint of LRMAHpan and Anthem

Two datasets containing multiple alleles were obtained from the IEDB database, presented by blood and breast cells. The blood dataset includes six HLA alleles, while the breast dataset has five alleles, lacking one HLA-A type. To meet the Anthem tool's requirements for peptide sequence length, we categorized the sequences into three lengths: 9, 10, and 11. Each category was stored in a separate file, and the ratio of presented peptides to non-presented peptides is 1:1. The number of peptide sequences for each length category is summarizedin the table below.

Presenting cell	HLA Types	Length	Presented peptides	non-presented peptides
Blood	A0101, A0301 B0702, B2705 C0202, C0702	9	6314	6314
		10	2992	2992
		11	1762	1762
Breast	A3101, B3508, B3701 C0401, C0602	9	4323	4323
		10	1235	1235
		11	998	998

Anthem Prediction: Predictions for each length category were made separately for the blood and breast datasets. This process generates the predicted scores for each HLA type corresponding to the peptide sequences. The results for the six HLA types were compiled into a single .csv file, Columns structured as following:

***Peptide**:* The peptide sequence being predicted

HLA-1 to HLA-6: Prediction scores for each HLA type presenting the peptide (scores range from 0 to 1, with higher values indicating a greater likelihood of presentation)

Best Predicted Score: The maximum score among HLA-1 to HLA-6

Label: The true values for each peptide

LRMAHpan Prediction with Incomplete HLA Typing Data: The breast dataset is missing one HLA-A type. To address this, we supplemented the prediction with data for the known HLA-A*31:01 type for that patient. Our model, LRMAHpan, showed strong predictive performance across AUC, AP, F1, and PPV metrics, regardless of missing HLA typing in the test dataset (see Figure S8). Furthermore, when peptide sequences were categorized by lengths (9–11 mers), LRMAHpan consistently outperformed Anthem.

Table S1 Key resources table

REAGENT or RESOURCE	SOURCE	IDENTIFIER
Deposited Data		
Training data: MA MS data	(Bulik-Sullivan et al. 2019), (O'Donnell, Rubinsteyn, and Laserson 2020)	https://github.com/openvax/mhcflurry
MULTIALLELIC benchmark dataset with predictions	(O'Donnell, Rubinsteyn, and Laserson 2020)	https://github.com/openvax/mhcflurry
MA pHLA-II	(Nielsen et al. 2010)	https://maria.stanford.edu/
Metastatic melanoma cohorts	(Gao et al. 2013)	http://www.cbioportal.org
Software and Algorithms		

LRMAHpan	this paper	https://github.com/Luckysoutheast/LRMAHpan.git
MHCflurry 2.0	(O'Donnell, Rubinsteyn, and Laserson 2020)	github.com/openvax/mhcflurry
NetMHCpan 4.1x	(Jurtz et al. 2017; Reynisson et al. 2020)	http://www.cbs.dtu.dk/services/ NetMHCpan-4.1
EDGE	(Bulik-Sullivan et al. 2019)	
MARIA	(Nielsen et al. 2010)	https://maria.stanford.edu/

Table S2 HLA Typings Capable of Presenting More than 30 Peptide Sequences in the Training Set

HLA-A1, HLA-A2, HLA- B1, HLA-B2, HLA-C1, HLA-C2
HLA-A*03:01,HLA-A*68:01,HLA-B*27:05,HLA-B*35:03,HLA-C*02:02,HLA-C*04:01
HLA-A*02:20,HLA-A*68:01,HLA-B*35:03,HLA-B*39:01,HLA-C*04:01,HLA-C*07:02
HLA-A*24:02,HLA-A*01:01,HLA-B*08:01,HLA-B*35:01,HLA-C*07:01,HLA-C*04:01
HLA-A*01:01,HLA-A*26:01,HLA-B*18:01,HLA-B*15:01,HLA-C*03:03,HLA-C*07:01
HLA-A*24:02,HLA-A*25:01,HLA-B*18:01,HLA-B*07:02,HLA-C*07:02,HLA-C*12:03
HLA-A*29:02,HLA-A*02:01,HLA-B*44:03,HLA-B*44:02,HLA-C*05:01,HLA-C*16:01
HLA-A*02:01,HLA-A*68:01,HLA-B*44:02,HLA-B*44:02,HLA-C*05:01,HLA-C*07:04
HLA-A*01:01,HLA-A*24:02,HLA-B*38:01,HLA-B*44:03,HLA-C*06:02,HLA-C*12:03
HLA-A*01:01,HLA-A*24:02,HLA-B*07:02,HLA-B*08:01,HLA-C*07:01,HLA-C*07:02
HLA-A*01:01,HLA-A*24:02,HLA-B*13:02,HLA-B*39:06,HLA-C*06:02,HLA-C*12:03
HLA-A*31:01,HLA-A*02:01,HLA-B*44:02,HLA-B*27:05,HLA-C*05:01,HLA-C*02:02
HLA-A*03:02,HLA-A*26:01,HLA-B*44:03,HLA-B*35:02,HLA-C*03:03,HLA-C*16:01
HLA-A*03:01,HLA-A*03:01,HLA-B*35:03,HLA-B*51:01,HLA-C*03:03,HLA-C*04:01
HLA-A*29:02,HLA-A*29:02,HLA-B*44:03,HLA-B*35:01,HLA-C*04:01,HLA-C*16:01
HLA-A*31:01,HLA-A*03:01,HLA-B*08:01,HLA-B*38:01,HLA-C*12:03,HLA-C*07:01
HLA-A*01:01,HLA-A*03:01,HLA-B*08:01,HLA-B*35:01,HLA-C*07:01,HLA-C*04:01
HLA-A*11:01,HLA-A*23:01,HLA-B*35:01,HLA-B*49:01,HLA-C*07:01,HLA-C*04:01
HLA-A*29:02,HLA-A*03:01,HLA-B*44:03,HLA-B*07:02,HLA-C*07:02,HLA-C*16:01
HLA-A*02:01,HLA-A*02:01,HLA-B*44:02,HLA-B*44:02,HLA-C*05:01,HLA-C*05:01
HLA-A*02:01,HLA-A*02:01,HLA-B*39:06,HLA-B*39:06,HLA-C*07:02,HLA-C*07:02
HLA-A*68:01,HLA-A*02:01,HLA-B*13:02,HLA-B*35:03,HLA-C*06:02,HLA-C*04:01
HLA-A*01:01,HLA-A*23:01,HLA-B*07:02,HLA-B*15:01,HLA-C*12:03,HLA-C*14:02
HLA-A*31:01,HLA-A*01:01,HLA-B*44:02,HLA-B*67:01,HLA-C*05:01,HLA-C*12:03
HLA-A*01:01,HLA-A*33:01,HLA-B*44:03,HLA-B*35:01,HLA-C*02:02,HLA-C*04:01
HLA-A*01:01,HLA-A*24:02,HLA-B*08:01,HLA-B*18:01,HLA-C*07:01,HLA-C*07:01
HLA-A*24:02,HLA-A*26:01,HLA-B*38:01,HLA-B*44:02,HLA-C*05:01,HLA-C*12:03
HLA-A*01:01,HLA-A*03:01,HLA-B*18:01,HLA-B*55:01,HLA-C*03:03,HLA-C*07:01
HLA-A*03:01,HLA-A*03:01,HLA-B*35:01,HLA-B*35:01,HLA-C*04:01,HLA-C*04:01
HLA-A*01:01,HLA-A*01:01,HLA-B*57:01,HLA-B*57:01,HLA-C*06:02,HLA-C*06:02
HLA-A*02:05,HLA-A*03:01,HLA-B*50:01,HLA-B*27:05,HLA-C*02:02,HLA-C*06:02

HLA-A*01:01,HLA-A*25:01,HLA-B*08:01,HLA-B*18:01,HLA-C*12:03,HLA-C*07:01
HLA-A*33:01,HLA-A*03:01,HLA-B*35:01,HLA-B*14:01,HLA-C*04:01,HLA-C*08:02
HLA-A*02:01,HLA-A*02:01,HLA-B*18:01,HLA-B*38:01,HLA-C*05:01,HLA-C*05:01
HLA-A*01:01,HLA-A*11:01,HLA-B*08:01,HLA-B*40:01,HLA-C*03:04,HLA-C*07:01
HLA-A*01:01,HLA-A*24:02,HLA-B*08:01,HLA-B*08:01,HLA-C*07:01,HLA-C*07:01
HLA-A*31:01,HLA-A*01:01,HLA-B*07:02,HLA-B*40:01,HLA-C*07:02,HLA-C*03:04
HLA-A*02:01,HLA-A*24:02,HLA-B*48:01,HLA-B*38:01,HLA-C*03:04,HLA-C*08:01
HLA-A*31:01,HLA-A*26:01,HLA-B*44:02,HLA-B*38:01,HLA-C*12:03,HLA-C*05:01
HLA-A*03:01,HLA-A*23:01,HLA-B*08:01,HLA-B*15:134,HLA-C*07:02,HLA-C*07:04
HLA-A*02:01,HLA-A*24:02,HLA-B*15:11,HLA-B*35:01,HLA-C*03:03,HLA-C*03:03
HLA-A*01:01,HLA-A*03:01,HLA-B*15:01,HLA-B*37:01,HLA-C*03:03,HLA-C*06:02
HLA-A*30:01,HLA-A*68:02,HLA-B*15:03,HLA-B*15:10,HLA-C*02:02,HLA-C*03:04
HLA-A*03:01,HLA-A*29:02,HLA-B*44:02,HLA-B*44:03,HLA-C*12:03,HLA-C*16:01
HLA-A*02:05,HLA-A*24:02,HLA-B*15:01,HLA-B*50:01,HLA-C*03:03,HLA-C*06:02
HLA-A*02:01,HLA-A*32:01,HLA-B*18:01,HLA-B*15:01,HLA-C*07:04,HLA-C*04:01
HLA-A*02:03,HLA-A*68:01,HLA-B*15:02,HLA-B*15:13,HLA-C*08:01,HLA-C*08:01
HLA-A*03:01,HLA-A*03:01,HLA-B*07:02,HLA-B*07:02,HLA-C*07:01,HLA-C*07:01
HLA-A*03:01,HLA-A*03:01,HLA-B*07:02,HLA-B*51:01,HLA-C*07:02,HLA-C*01:02
HLA-A*30:01,HLA-A*24:02,HLA-B*13:02,HLA-B*40:02,HLA-C*03:04,HLA-C*06:02
HLA-A*01:01,HLA-A*32:01,HLA-B*15:01,HLA-B*15:01,HLA-C*03:03,HLA-C*03:04
HLA-A*01:01,HLA-A*11:01,HLA-B*08:01,HLA-B*55:01,HLA-C*03:03,HLA-C*07:01
HLA-A*23:01,HLA-A*01:01,HLA-B*08:01,HLA-B*15:03,HLA-C*01:02,HLA-C*12:03
HLA-A*01:01,HLA-A*32:01,HLA-B*13:02,HLA-B*40:02,HLA-C*02:02,HLA-C*06:02
HLA-A*02:01,HLA-A*03:01,HLA-B*07:02,HLA-B*07:02,HLA-C*07:02,HLA-C*07:02
HLA-A*01:01,HLA-A*03:01,HLA-B*07:02,HLA-B*08:01,HLA-C*07:01,HLA-C*07:02
HLA-A*23:01,HLA-A*24:02,HLA-B*07:02,HLA-B*40:01,HLA-C*03:04,HLA-C*07:02
HLA-A*02:01,HLA-A*24:02,HLA-B*15:01,HLA-B*73:01,HLA-C*03:03,HLA-C*15:05
HLA-A*01:01,HLA-A*01:01,HLA-B*08:01,HLA-B*08:01,HLA-C*07:01,HLA-C*07:01
HLA-A*02:01,HLA-A*02:01,HLA-B*07:02,HLA-B*07:02,HLA-C*07:02,HLA-C*07:02
HLA-A*02:01,HLA-A*11:01,HLA-B*44:02,HLA-B*44:03,HLA-C*05:01,HLA-C*16:01
HLA-A*02:01,HLA-A*26:01,HLA-B*40:01,HLA-B*44:02,HLA-C*03:04,HLA-C*05:01
HLA-A*02:01,HLA-A*02:02,HLA-B*13:02,HLA-B*40:02,HLA-C*02:02,HLA-C*06:02
HLA-A*02:01,HLA-A*24:02,HLA-B*15:01,HLA-B*18:01,HLA-C*03:03,HLA-C*07:01
HLA-A*32:01,HLA-A*23:01,HLA-B*44:03,HLA-B*44:06,HLA-C*05:01,HLA-C*04:01
HLA-A*11:01,HLA-A*68:01,HLA-B*51:01,HLA-B*56:01,HLA-C*01:02,HLA-C*07:02
HLA-A*01:01,HLA-A*03:01,HLA-B*08:01,HLA-B*18:01,HLA-C*07:01,HLA-C*07:01
HLA-A*11:01,HLA-A*11:01,HLA-B*27:02,HLA-B*55:01,HLA-C*03:03,HLA-C*05:01
HLA-A*03:01,HLA-A*24:02,HLA-B*44:03,HLA-B*55:01,HLA-C*03:03,HLA-C*16:01
HLA-A*01:01,HLA-A*02:01,HLA-B*07:02,HLA-B*08:01,HLA-C*07:01,HLA-C*07:02
HLA-A*29:01,HLA-A*02:01,HLA-B*35:01,HLA-B*51:01,HLA-C*04:01,HLA-C*15:02
HLA-A*02:01,HLA-A*02:01,HLA-B*07:02,HLA-B*44:02,HLA-C*07:02,HLA-C*07:02
HLA-A*02:01,HLA-A*01:01,HLA-B*08:01,HLA-B*15:01,HLA-C*03:04,HLA-C*07:01
HLA-A*31:01,HLA-A*31:01,HLA-B*35:08,HLA-B*37:01,HLA-C*04:01,HLA-C*06:02

HLA-A*01:01,HLA-A*02:01,HLA-B*18:01,HLA-B*39:03,HLA-C*05:01,HLA-C*07:01
HLA-A*33:03,HLA-A*68:01,HLA-B*44:03,HLA-B*35:01,HLA-C*07:01,HLA-C*04:01
HLA-A*02:01,HLA-A*11:01,HLA-B*40:01,HLA-B*40:01,HLA-C*03:03,HLA-C*03:03
HLA-A*01:01,HLA-A*01:01,HLA-B*38:01,HLA-B*56:01,HLA-C*01:02,HLA-C*06:02
HLA-A*01:01,HLA-A*03:01,HLA-B*08:01,HLA-B*07:02,HLA-C*07:02,HLA-C*07:01
HLA-A*03:01,HLA-A*03:01,HLA-B*35:01,HLA-B*15:01,HLA-C*03:03,HLA-C*04:01
HLA-A*01:01,HLA-A*02:01,HLA-B*07:02,HLA-B*44:03,HLA-C*07:02,HLA-C*16:01
HLA-A*02:01,HLA-A*29:02,HLA-B*44:03,HLA-B*57:01,HLA-C*07:01,HLA-C*16:01
HLA-A*01:01,HLA-A*02:01,HLA-B*45:01,HLA-B*18:01,HLA-C*05:01,HLA-C*07:01
HLA-A*02:01,HLA-A*02:01,HLA-B*07:02,HLA-B*44:02,HLA-C*05:01,HLA-C*07:02
HLA-A*24:02,HLA-A*23:01,HLA-B*51:01,HLA-B*15:01,HLA-C*03:03,HLA-C*14:02
HLA-A*03:01,HLA-A*24:02,HLA-B*14:01,HLA-B*51:01,HLA-C*01:02,HLA-C*08:02
HLA-A*30:02,HLA-A*33:03,HLA-B*08:01,HLA-B*35:01,HLA-C*07:01,HLA-C*07:01
HLA-A*02:06,HLA-A*24:02,HLA-B*07:02,HLA-B*55:01,HLA-C*01:02,HLA-C*07:02
HLA-A*03:01,HLA-A*29:02,HLA-B*08:01,HLA-B*44:03,HLA-C*07:01,HLA-C*16:01
HLA-A*02:01,HLA-A*26:01,HLA-B*14:01,HLA-B*38:01,HLA-C*08:02,HLA-C*12:03
HLA-A*02:01,HLA-A*03:01,HLA-B*07:02,HLA-B*14:01,HLA-C*07:02,HLA-C*08:02
HLA-A*32:01,HLA-A*03:01,HLA-B*35:01,HLA-B*27:05,HLA-C*02:02,HLA-C*04:01
HLA-A*03:01,HLA-A*11:01,HLA-B*44:03,HLA-B*50:01,HLA-C*07:02,HLA-C*07:01
HLA-A*02:01,HLA-A*03:01,HLA-B*13:02,HLA-B*47:01,HLA-C*06:02,HLA-C*06:02
HLA-A*01:01,HLA-A*03:01,HLA-B*07:02,HLA-B*27:05,HLA-C*02:02,HLA-C*07:01
HLA-A*01:01,HLA-A*32:01,HLA-B*08:01,HLA-B*08:01,HLA-C*07:02,HLA-C*07:01
HLA-A*02:01,HLA-A*23:01,HLA-B*18:01,HLA-B*15:03,HLA-C*02:02,HLA-C*02:02
HLA-A*02:01,HLA-A*03:01,HLA-B*27:05,HLA-B*47:01,HLA-C*01:02,HLA-C*06:02
HLA-A*02:01,HLA-A*24:02,HLA-B*35:03,HLA-B*44:02,HLA-C*05:01,HLA-C*12:03
HLA-A*02:01,HLA-A*32:01,HLA-B*44:02,HLA-B*52:01,HLA-C*12:02,HLA-C*16:04
HLA-A*02:01,HLA-A*03:01,HLA-B*44:02,HLA-B*07:02,HLA-C*07:02,HLA-C*05:01
HLA-A*31:01,HLA-A*02:05,HLA-B*44:03,HLA-B*49:01,HLA-C*07:01,HLA-C*04:01
HLA-A*03:01,HLA-A*24:02,HLA-B*07:02,HLA-B*55:01,HLA-C*07:02,HLA-C*03:03
HLA-A*02:01,HLA-A*23:01,HLA-B*18:01,HLA-B*44:03,HLA-C*07:02,HLA-C*07:01
HLA-A*32:01,HLA-A*26:01,HLA-B*45:01,HLA-B*44:02,HLA-C*05:01,HLA-C*06:02
HLA-A*02:01,HLA-A*11:01,HLA-B*40:01,HLA-B*44:03,HLA-C*07:02,HLA-C*07:01
HLA-A*11:01,HLA-A*11:01,HLA-B*18:03,HLA-B*35:01,HLA-C*07:02,HLA-C*07:01
HLA-A*11:01,HLA-A*11:01,HLA-B*14:01,HLA-B*44:02,HLA-C*07:02,HLA-C*07:01
HLA-A*11:01,HLA-A*11:01,HLA-B*14:01,HLA-B*44:02,HLA-C*12:03,HLA-C*14:02
HLA-A*30:04,HLA-A*33:01,HLA-B*14:01,HLA-B*49:01,HLA-C*07:01,HLA-C*08:02
HLA-A*11:01,HLA-A*11:01,HLA-B*18:03,HLA-B*35:01,HLA-C*07:02,HLA-C*16:01
HLA-A*02:01,HLA-A*11:01,HLA-B*27:05,HLA-B*56:01,HLA-C*07:02,HLA-C*07:01
HLA-A*03:01,HLA-A*32:01,HLA-B*27:05,HLA-B*45:01,HLA-C*07:02,HLA-C*07:01
HLA-A*02:01,HLA-A*24:02,HLA-B*15:01,HLA-B*44:02,HLA-C*05:01,HLA-C*07:02
HLA-A*03:01,HLA-A*31:01,HLA-B*14:01,HLA-B*35:02,HLA-C*04:01,HLA-C*08:02
HLA-A*02:01,HLA-A*02:01,HLA-B*13:02,HLA-B*41:01,HLA-C*07:02,HLA-C*07:01
HLA-A*02:01,HLA-A*02:01,HLA-B*13:02,HLA-B*41:01,HLA-C*07:02,HLA-C*08:02

HLA-A*03:01,HLA-A*24:02,HLA-B*07:02,HLA-B*27:05,HLA-C*07:02,HLA-C*07:01
HLA-A*02:06,HLA-A*24:02,HLA-B*08:01,HLA-B*51:01,HLA-C*07:02,HLA-C*14:02
HLA-A*03:01,HLA-A*68:01,HLA-B*27:05,HLA-B*35:03,HLA-C*02:02,HLA-C*04:01
